# Ecotype Diversity and Conversion in *Photobacterium profundum* Strains

**DOI:** 10.1371/journal.pone.0096953

**Published:** 2014-05-13

**Authors:** Federico M. Lauro, Emiley A. Eloe-Fadrosh, Taylor K. S. Richter, Nicola Vitulo, Steven Ferriera, Justin H. Johnson, Douglas H. Bartlett

**Affiliations:** 1 School of Biotechnology and Biomolecular Sciences, The University of New South Wales, Sydney, New South Wales, Australia; 2 Singapore Centre on Environmental Life Sciences Engineering (SCELSE), Nanyang Technological University, Singapore; 3 Marine Biology Research Division, Scripps Institution of Oceanography, University of California San Diego, La Jolla, California, United States of America; 4 CRIBI Biotechnology Centre, University of Padua, Padova, Italy; 5 J. Craig Venter Institute, Rockville, Maryland, United States of America; Oak Ridge National Lab, United States of America

## Abstract

*Photobacterium profundum* is a cosmopolitan marine bacterium capable of growth at low temperature and high hydrostatic pressure. Multiple strains of *P. profundum* have been isolated from different depths of the ocean and display remarkable differences in their physiological responses to pressure. The genome sequence of the deep-sea piezopsychrophilic strain *Photobacterium profundum* SS9 has provided some clues regarding the genetic features required for growth in the deep sea. The sequenced genome of *Photobacterium profundum* strain 3TCK, a non-piezophilic strain isolated from a shallow-water environment, is now available and its analysis expands the identification of unique genomic features that correlate to environmental differences and define the Hutchinsonian niche of each strain. These differences range from variations in gene content to specific gene sequences under positive selection. Genome plasticity between *Photobacterium* bathytypes was investigated when strain 3TCK-specific genes involved in photorepair were introduced to SS9, demonstrating that horizontal gene transfer can provide a mechanism for rapid colonisation of new environments.

## Introduction

The vast majority of the earth’s marine biosphere is at a relatively constant low temperature, high hydrostatic pressure and is constrained by the small amounts of refractory organic nutrients that arrive in pulses from the overlying photic zone [1]. These conditions promote and maintain a diverse microbial community as detected by culture-independent approaches [2], [3], [4]. It is still under debate the extent to which the culture-independent diversity is autochthonous [4], but generally pressure-adaptation is considered a valid criterion to discriminate against microbes recently introduced to the deep sea from shallower waters [4], [5], [6]. With few exceptions [6], [7], [8], [9] the majority of pressure-adapted isolates in culture span only a narrow phylogenetic range of gamma proteobacteria [10]. This includes the easily culturable *Photobacterium profundum* SS9 that has become a model for studying adaptations to high pressure.

With the completion of the *P. profundum* SS9 genome sequence [11], the details of physiological responses to pressure have begun to unravel: for example, microarray studies have shown that suboptimal hydrostatic pressure induces the up-regulation of chaperones and DNA repair enzymes [12], and RNA-seq analyses have revealed the differential expression of multiple ATP synthases and membrane transporters [13].

At least four separate strains of *P. profundum* (SS9, DSJ4, 3TCK and 1230sf1) have been isolated and characterized from multiple sites in the Pacific ocean [10], [14], [15], [16]. While strains SS9 and DSJ4 were isolated from deep-sea environments and are adapted to high hydrostatic pressure [14], [15], strains 1230sf1 and 3TCK were recovered from shallower waters and are inhibited by elevated hydrostatic pressure (12 and our unpublished results). These different ecotypes, which vary in their adaptation to depth in the water column, have been defined bathytypes [5]. The very existence of phylogenetically cohesive bathytypes suggests that the genetic modifications required to evolve depth-specific adaptations can be rapidly evolved [10] and bathytype conversion might occur quite frequently as a result of advective transport of microbial communities by phenomena such as up/downwelling, Ekman transport and thermohaline circulation [10], [17]. The rapid development of mutants tolerant to pressure inactivation, which has been found to extend into the gigapascal range even for non-marine bacteria [18], might aid specific taxa in rapidly adapting to the new environmental conditions while restricting others.

In this study the genome plasticity between two bathytypes of *P. profundum* is analysed in detail. The results show that no single gene is likely to restrict the environmental niche (sensu Hutchinson [19]) of each strain, but a number of genetic features specific to each strain can confer specific abilities to cope with depth-specific environmental stresses (e.g. temperature, pressure, nutrient availability). Some of these features carry signatures of horizontal gene transfer (HGT) suggesting one possible mechanism for the rapid evolution of new bathytypes.

To test the feasibility of bathytype conversion, a cluster of genes involved in the repair of ultraviolet light-induced DNA damage were transferred from the shallow to the deep bathytype. The lack of this UV protective function is predicted to restrict the colonization of shallower waters by deep bathytypes. This is the first study employing intra-specific sequence comparisons in combination with molecular genetics to address the bases of niche partitioning in piezophilic bacteria.

## Materials and Methods

### Strains and Growth Conditions

The bacterial strains used in this study are listed in Table 1. The strains of *P. profundum* were cultured in 75% strength 2216 Marine Medium (28 g/l; Difco Laboratories) at 15°C and 0.1 megapascal (MPa), unless otherwise specified. *E. coli* strains were grown aerobically at 37°C in Luria-Bertani (LB) medium. High-pressure growth experiments were performed by inoculating in heat-sealable plastic bulbs containing media and no gas space. The heat-sealed bulbs were placed in pressure vessels and pressurized as previously described [20], [21].

**Table 1:** Strains and Plasmids used in this study.

Strain/plasmid	Relevant genotype or description	Reference
*P. profundum*		
SS9	Wild type, deep bathytype of *P. profundum*	[14], [15]
DSJ4	Wild type, deep bathytype of *P. profundum*	[15]
3TCK	Wild type, shallow bathytype of *P. profundum*	[12]
SS9R	Rif^r^ SS9 derivative	[22]
*** ***3TCKR	Rif^r^ 3TCK derivative	This study
*E. coli* strains		
ED8654	pRK2073 maintenance	[60]
DH5α	*recA^−^*, used for cloning	[61]
XL1-Blue	*recA^−^*, used for cloning	Stratagene
TOP10	*recA^−^*, used for cloning	Invitrogen
Plasmids		
pRK2073	Providing *tra* genes for conjugal transfer	[62]
pFL122	RSF1010 derived, broad host range cloning vector, Sm^r^	[37]
pFL190	RSF1010 derived, arabinose-inducible expression vector, Sm^r^	[37]
pFL303	*phr* gene cluster in pFL122, Sm^r^	This study
pFL304	Δ22 deletion of *phr* gene cluster in pFL122, Sm^r^	This study
pFL305	pFL304+*phr* promoter, Sm^r^	This study
pFL306	pFL304+*rpoX*+*phr* promoter, Sm^r^	This study
pFL307	*phr* in pFL190, Sm^r^	This study

When needed, antibiotics were used in the following final concentrations: rifampicin (Rif), 100 µg/ml; kanamycin (Km), 100 µg/ml (*E. coli*) or 200 µg/ml (*P. profundum*); streptomycin (Sm), 50 µg/ml (*E. coli*) or 150 µg/ml (*P. profundum*). X-Gal (5-bromo-4-chloro-3-indolyl- [beta]-D-galactopyranoside) was added to solid medium at 40 µg/ml in N,N-dimethylformamide. The introduction of plasmids in *P. profundum* was achieved by tri-parental conjugations using the helper *E. coli* strain pRK2073 as previously described [22].

### Genome Sequencing, Assembly and Annotation

Genomic DNA was obtained from a culture of *P. profundum* strain 3TCK in mid-exponential growth. Approximately 1 liter of a liquid culture was harvested by centrifugation for 15 minutes at 5,000×g and the pellet was resuspended in 5 ml buffer A (50 mM Tris, 50 mM EDTA, pH 8.0). The suspension was incubated overnight at −20°C and thawed at room temperature with the addition of 500 µl of buffer B (250 mM Tris, pH 8.0, 10 mg/ml lysozime). After 45 min of incubation on ice, 1 ml of buffer C (0.5% SDS, 50 mM Tris, 400 mM EDTA, pH 7.5, 1 mg/ml Proteinase K) was added and the mixture was placed in a 50°C water bath for 60 minutes. An additional 750 µl of buffer C were added followed by an additional 30 minutes of incubation at 50°C. The genomic DNA was extracted twice with 5 ml of phenol:chloroform:isoamyl alcohol (24∶24∶1), and precipitated with 0.8 volumes of isopropanol. The DNA pellet was recovered by spooling on a glass rod, and rehydrated overnight at 4°C in 4 ml of buffer D (50 mM Tris, 1 mM EDTA, 200 ug/ml RNAse A, pH 8.0). Further purification was performed by extracting once with 4 ml of chloroform, then precipitating with 3.2 ml of isopropanol. The DNA pellet was recovered by centrifugation, washed once with 5 ml of 70% ethanol and stored dry at −20°C.

The dry DNA pellet was shipped to the J. Craig Venter Institute where a draft genome sequence was obtained with a conventional whole-genome sequencing approach by preparing two genomic libraries with insert sizes of 4 kb and 40 kb as described in Goldberg et al. [23] and the resulting sequences were used as input for the Celera assembler [24]. The draft genome sequence was deposited in NCBI under the BioProject accession number PRJNA13563. The reference genome sequence of *P. profundum* SS9 was also retrieved from NCBI under BioProject accession number PRJNA13128. The number of ribosomal RNA operons was estimated by Pulsed Field Gel Electrophoresis of genomic DNA digested with I-*Ceu*I as previously described [25]. The draft assembly was submitted to the NCBI PGAAP (http://www.ncbi.nlm.nih.gov/genomes/static/Pipeline.html) for prediction of Open Reading Frames (ORFs) and automatic annotation. For genomic sequence comparisons the scaffolds of 3TCK were oriented and joined in alignments to the reference genome of SS9 with the 6-frame stop-codon spacer ‘NNNNCACACACTTAATTAATTAAGTGTGTGNNNN’ using the custom perl script scaffolding.pl to create a contiguous pseudomolecule. Scaffolding.pl and other custom perl scripts used in this study are available at https://github.com/flauro/3tck_comparative.

### Bioinformatic Analyses

The assignment of ORFs to Clusters of Orthologous Groups (COGs) and statistical comparisons were performed as previously described [26] using the method of Rodriguez-Brito [27] with a subsample size of 4000 and 10,000 bootstraps. The average nucleotide identity (ANI) between the genomes was computed as a reciprocal two-way average with the method of Goris et al. [28] using custom perl scripts and the following parameters: fragment size 1020 bp; minimum identity 30%; minimum alignable region 714 bp.

The ratio of non-synonymous substitutions per non-synonymous site to synonymous substitutions per synonymous site (ω) was computed for every pair of orthologous genes using a custom perl pipeline. Briefly, orthologous gene-pairs were found using the reciprocal smallest distance algorithm [29], aligned with MUSCLE [30], and ω was calculated using the KaKs calculator [31] with the YN00 method [32]. The statistical significance of orthologous pairs with ω>1 was assessed with a Fisher exact test. The time of divergence between the strains (τ) was estimated from the formula τ = Ks/(2*λ) where λ = 8.3×10^−7^ SNPs/site/year [33] medianed across all pairs of orthologs. Codon usage was calculated as described by Karlin [34] using a custom perl script. The relevant biases in codon usage were identified using the methods described in [35], [36]. Briefly, the codon usage of each gene is compared to the genome-wide mode of codon usage, and the significance is established using a Chi-square test. The p-value threshold was set to 0.1.

### Cloning Experiments

All restriction enzymes were purchased from New England Biolabs (Beverly, MA, USA). All the PCR amplifications were performed using the Expand Long Template PCR system (Roche Applied Science, Indianapolis, IN, USA).

The genes conferring UV resistance were cloned in pFL122 as follows. A fosmid clone (GCLNU_G4) from the *P. profundum* 3TCK sequencing library containing P3TCK_10673 (*rpoX*; RNA polymerase sigma factor, ECF subfamily), P3TCK_10668 (Conserved Hypothetical Protein), P3TCK_10663 (*phr*; deoxyribodipyrimidine photolyase) was cut with *Xho*I+*Kpn*I. The 7.2 kbp band contained the genes of interest and was gel purified and ligated in pFL122 [37] cut with *Xho*I+*Kpn*I yielding pFL303. The deletion Δ22 was obtained by cutting pFL303 with *Eco*RI and re-ligating, which effectively eliminates P3TCK_10673, most of P3TCK_10668 and the region with the two divergent promoters between the two. This deletion construct was named pFL304. The promoter region was PCR amplified from pFL303 using primers PROMPHO2F (5′ – GTCGAATTCCTTTTCTTGCAGCGTCAGT - 3′) and PROMPHO2R (5′ – GTCGAATTCTAGTAAGCGAATAGCAGGAC -3′).

Similarly the promoter region and the whole length P3TCK_10673 was amplified with primers SIGMAPHO2F (5′ – GTCGAATTCGTATTCAAGATGGGCACTCA – 3′) and the same reverse primer as above. Both amplicons were digested with *Eco*RI and cloned in the *Eco*RI site of pFL304 yielding pFL305 (promoter only) and pFL306 (promoter and P3TCK_10673) respectively. The directionality of the inserts was checked by PCR and confirmed by standard thermal cycle dideoxy sequencing with fluorescently labelled terminators (Applied Biosystems, Foster City, CA, USA).

For the arabinose-inducible UV resistance experiments, the *phr* gene, inclusive of its ribosome binding site, was amplified with primers expPHO2F (5′ – ATGGCCGTCTGCAAGATCCTGTA -3′) and expPHO2R (5′ – GCTCTAGAGCCACCCATTCATACGATGTGC – 3′), digested with *Eco*RI+*Xba*I and cloned in the expression vector pFL190 [37] cut with the same enzymes.

### *In vivo* Photoreactivation

The effect of ultraviolet light on the survival of *P. profundum* strains was tested as follows. Serial dilutions of late exponential cultures were plated on 75% strength 2216 Marine Agar. For each strain a triplicate dilution series was prepared: one untreated, one UV irradiated without blue light recovery, and one UV irradiated followed by a recovery period under blue light.

The cells were irradiated uncovered using a germicidal lamp with an emission peak at 253.7 nm (Philips G25 T8), for 10 seconds at a power of 220 µW/cm2.

For the photoreactivation, the Petri dishes were then covered, to filter out the shorter wavelength radiation, and allowed to recover for 1 hour under “black” light (Philips TLD 15 W/08), emitting in the 350–400 nm range, at an irradiance of 20 µW/cm2. Irradiances were measured with a Spectroline DM-365 XA digital radiometer (Spectronics corp., Westbury, NY, USA).

The plates were wrapped in foil and grown at 15°C for 5 days after which c.f.u. were counted and the number of colonies in the irradiated samples were compared with the untreated controls to calculate the percent survival.

In all experiments, cell transfers and manipulations were performed under General Electric “gold” fluorescent light to prevent uncontrolled photorepair.

## Results and Discussion

Bacteria and Archaea can be transported vertically through the water column as a result of attachment to sinking particles (see, for example [38]) and migrating metazoans or other phenomena such as advective transport [17]. Growth and survival at different depths requires adaptation to many depth-correlated chemo-physical parameters (e.g. light, hydrostatic pressure, organic carbon). For example, it has been shown that adapting to a higher hydrostatic pressure requires adjustments to membrane structure, DNA synthesis, translation, and protein quaternary structure [39]. Pressure also affects gene regulation at the level of transcription [12], [13] and translation [40]. The concentration of nutrients varies greatly as a function of depth and it is possible that marine bacteria use pressure as a proxy for depth in gene regulation to respond to differences in nutrient availability. The switch between different outer membrane porins as a function of pressure, which has been observed in *P. profundum* SS9, is likely a result of such a response. Conversely, adaptation to shallow waters would require the acquisition of novel genes to cope with unique stressors, such as UV light. Many of these features are evident from the genome comparisons of different bathytypes of *P. profundum* presented here.

### General Features of the Draft Genome of *Photobacterium profundum* Strain 3TCK and Comparisons with the Genome of the Previously Sequenced Strain SS9

The draft genome of the shallow bathytype *Photobacterium profundum* 3TCK contains 11 scaffolds for a total length of 6,186,725 bp with average 41.3% GC encoding for a total of 5549 ORFs. Gene synteny plots and the existence of two different origins of replication [41] suggest that, similar to other members of the family Vibrionaceace [42], the genome is organised in two chromosomes. This size and structure is comparable to that of the previously sequenced deep bathytytpe SS9 [11], but appears to lack an 80 kb dispensable plasmid specific to SS9 [12].

The genome encodes for a complete set of tRNA synthetases and shares with SS9 the peculiarity of having the genes for the synthesis of selenocysteine and its incorporation into proteins. Like SS9 the genome also encodes for two complete F_0_F_1_-ATP-synthases and a type A FAS/PKS system [43] for the synthesis of polyunsaturated fatty acids such as eicosapentaenoic acid (EPA; 20: 5n−3).

*P. profundum* 3TCK has larger-than-average intergenic regions (∼167 bp), a feature shared with most sequenced piezophiles [5], although the size of the intergenics is smaller than in the deep bathytype SS9 (∼205 bp). These large intergenic regions have been shown to be transcribed and differentially expressed as a function of pressure [13], suggesting they could play an important physiological role.

**Figure 1:**
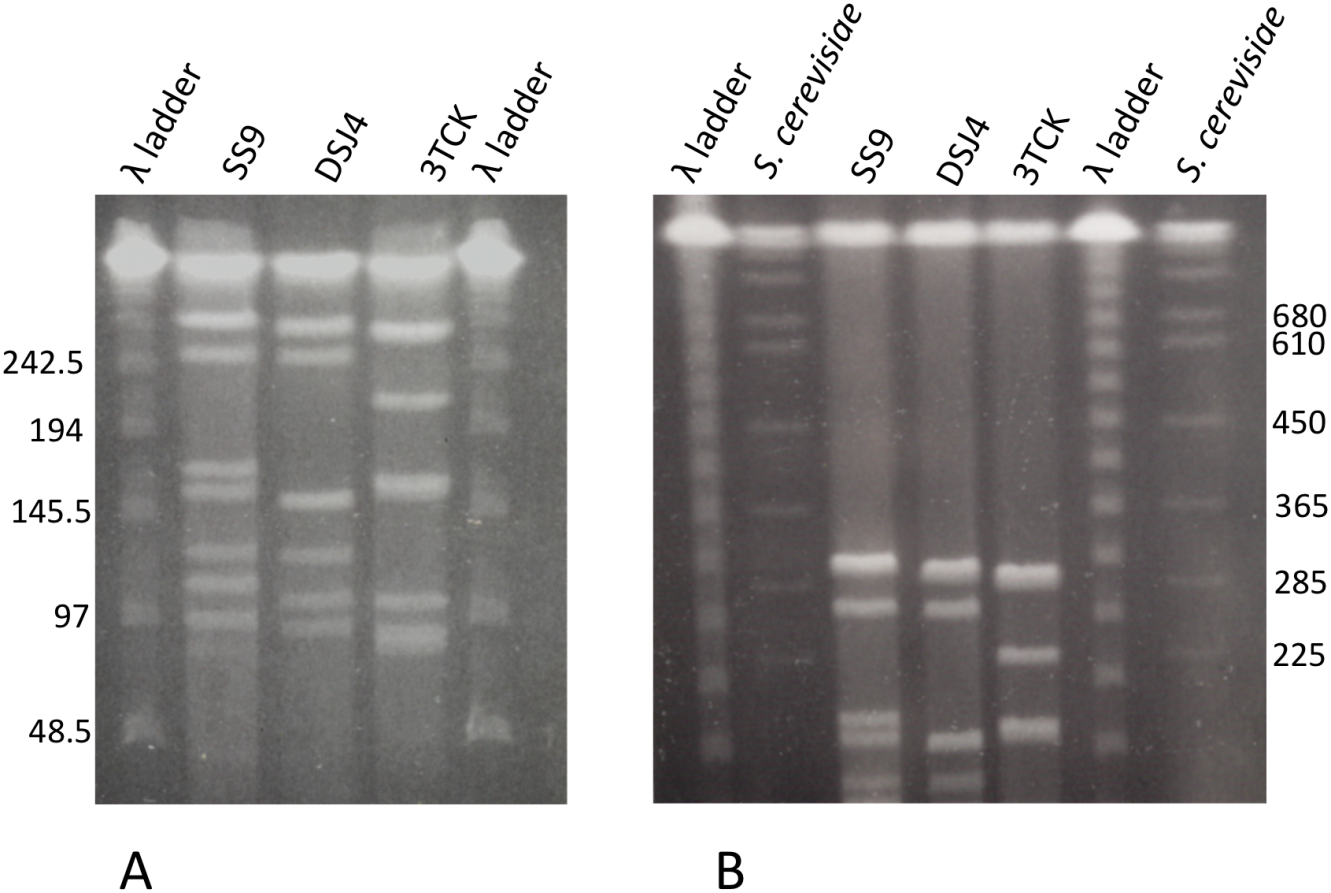
Pulsed field gel electrophoresis of chromosomal plugs of *P. profundum* strains SS9, DSJ4 and 3TCK. The plugs were digested overnight with *I-Ceu*I and run under the following conditions: (A) Voltage: 6 V/cm; Pulse-time: 3–18.5 s; Runtime: 18.5 h; Temperature: 14°C; Included angle: 120°; Gel: 0.95% PFGE agarose in 0.5x TBE (B) Voltage: 6 V/cm; Pulse-time: 6.7–54 s; Runtime: 28 h; Temperature: 14°C; Included angle: 120°; Gel: 0.95% PFGE agarose in 0.5x TBE.

*P. profundum* 3TCK contains at least 9 copies of the ribosomal RNA operon (rrn; Figure 1) a number which is larger than the median for microbial genomes [44]. Interestingly, SS9 still holds the record for the highest copy rrn number in a single genome with 15 copies. These operons display intragenomic variation in *P. profundum* SS9, while they are almost identical in 3TCK. The variability is concentrated in specific loops of the 16S and the 23S rRNA subunits [10], [45] and is predicted to contribute to the ribosome stability or function at high-hydrostatic pressure.

**Figure 2:**
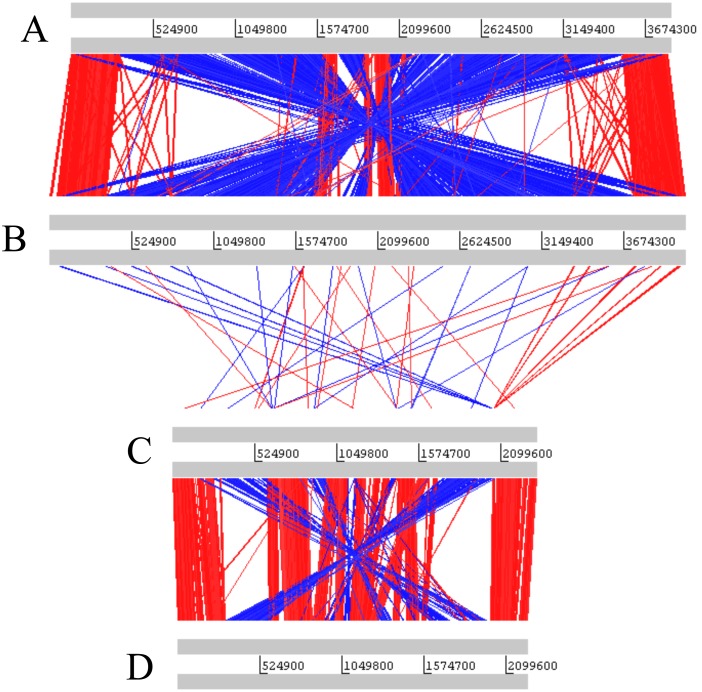
Chromosome organization in *P. profundum* strains. ACT nucleotide comparison [4] between the chromosomes of the two bathytypes SS9 and 3TCK. From top to bottom: (A) *P. profundum* 3TCK chromosome 1 (B) *P. profundum* SS9 chromosome 1 (C) *P. profundum* SS9 chromosome 2 (D) *P. profundum* 3TCK chromosome 2.

ACT comparisons [46] between the nucleotide sequences of the two strains highlights the presence of a number of insertions/deletions, multiple inversions across the origin/terminus of both chromosomes, but only a limited number of translocations across the chromosomes (Figure 2).

**Figure 3:**
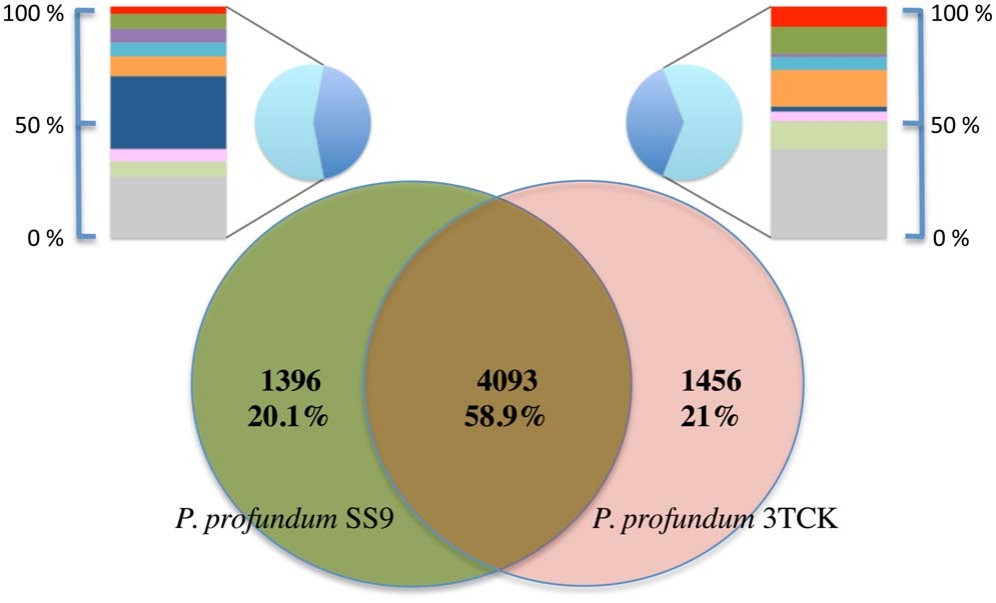
Gene content comparison in *P. profundum* strains. Shared gene comparison between the two bathytypes SS9 and 3TCK. The larger Venn diagram shows the number of orthologous genes shared and the number of genes unique to each bathytype. The smaller pie charts are the proportion of unique genes to each bathytype with (light blue) or without (dark blue) matches to the COG database. The matching genes were assigned to COG categories (from top to bottom): E-Amino acid transport and metabolism (red); G-Carbohydrate transport and metabolism (green); N-Motility and chemotaxis (violet); S-Function unknown (cyan); R-General function prediction only (orange); L-DNA Replication, Recombination and Repair (blue); T-Signal Transduction (pink); K-Transcription (light green); Other categories (grey).

The identity between the 16S gene sequences of 3TCK and SS9 is 98.73%, which suggests the strains belong to the same species, however the ANI between 3TCK and SS9 is 92.85% with a percent conserved DNA of 62.68%, which is well below the species threshold for genome-level comparisons (ANI>95%; conserved DNA>69%; [28]). The largest proportion of genes unique to each genome is located on chromosome 2 (Figure 2 and Figure 3). Within the family Vibrionaceae this chromosome has been previously implicated in gene capture for environmental adaptations during the colonization of new niches [42].

The global analysis of Ka/Ks ratios identified only four gene pairs with ω>1, but none of these was statistically significant determined by a Fisher exact test (P<0.01). The median time of divergence between the strains was 126,833 years ago which is remarkably comparable to the time of establishment of the modern thermohaline circulation. This network of advective currents is the predicted cause behind the spatial separation of the 2 bathytypes.

The extreme synteny, large proportion of insertions and deletions in association with a low number of sequences with ω>1 and the relatively short divergence time suggests a larger role for HGT rather than sequence substitutions in the evolution of bathytypes. Furthermore, it is a clear indication that the strains are currently undergoing adaptive radiation driven primarily by gene acquisition and loss.

**Figure 4:**
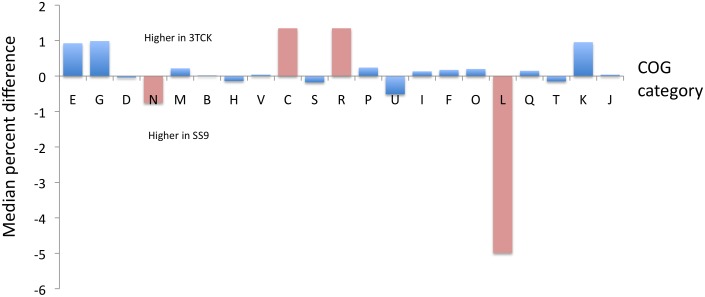
COG analysis comparison of the gene content in the two bathytypes. Reported are the median differences after resampling as described in material and methods. In pink the categories that were statistically over- or under-represented in one of the bathytypes at 98% significance.

COG comparisons between the two strains revealed a statistical over-representation in the shallow bathytype 3TCK of genes for energy production (COG category C,) but a significant decrease in genes for motility and chemotaxis (N) and DNA replication, recombination and repair (L) (Figure 4).

The abundance of genes for category L (DNA replication, recombination and repair) in the deep bathytype SS9 is due to the large number of transposable elements. The amplification of mobile elements seems to be a distinctive feature of all sequenced deep-sea genomes and has been observed in metagenomic surveys of different depths in the water column [3], [47]. The wide diversity of the identified transposases in the deep-sea samples that could not be accounted for by biases in community composition led DeLong et al. [47] to hypothesize that the over-representation of transposable elements relates to the slower growth and smaller effective population size of deep-sea microbial communities. Compatible with this hypothesis, the 206 COG-categorized transposable elements found in SS9 belong to 11 families, the most numerous of which (COG3436) has as many as 72 members. On the other hand, 3TCK encodes for just 3 COG-categorized transposable elements. Taken together these data support the hypothesis of intra-genomic amplification of transposable elements in the deep-sea due to habitat differences. Nevertheless, a transposon mutant with a cold-sensitive phenotype has been isolated in SS9 [48] suggesting that these mobile elements could have a functional role.

In *P. profundum* SS9 the genes for motility and flagellar assembly are arranged in two large clusters, one that is shared with 3TCK and a second one that is unique to the piezophile SS9 as a result of a large contiguous deletion in the genome of 3TCK. This deletion accounts for the under-representation of genes from COG category N (Motility and Chemotaxis) in the genome of 3TCK. This second cluster is most similar to a lateral flagella gene cluster present in some *Vibrio* strains [49] and its role in motility and chemotaxis has been previously analysed [50] comparing the piezosensitive motility of 3TCK to the piezoresistant one of SS9.

Another relevant genomic island encompasses genes PBPRA2666-PBPRA2712. This gene cluster is involved in cell-envelope biosynthesis and was initially suggested to be missing in 3TCK during a previous microarray study [12]. However, the genome sequence of 3TCK does, in fact, contain a similar and syntenic cluster (P3TCK_26512-P3TCK_26972), but with a highly divergent sequence. Interestingly, the cluster in SS9 contains at least 3 genes (PBPRA2678, PBPRA2681, PBPRA2684) that cause a cold-sensitive phenotype when inactivated [48], 8 genes that are regulated by temperature or pressure at the level of transcription (PBPRA2689, PBPRA2691, PBPRA2692, PBPRA2697, PBPRA2701, PBPRA2702, PBPRA2707, PBPRA2710) [12], [13] and 2 at the level of translation (PBPRA2686, PBPRA2687) [40].

Conversely, the over-representation in 3TCK of genes for Energy Production and Conversion (C) is caused by the expansion of COGs involved in anaerobic respiration of nitrate such as the periplasmic nitrate reductase napABCEG (COG0243, COG3043, COG3005, COG4459, COG1145), formate dehydrogenase (COG1526), cytochrome c553 (COG2863) and a two-component response regulator (COG2197) specific for nitrate reduction (P3TCK_05837). In addition chromosome 2 of 3TCK contained a 12 kb genomic island encompassing ORFs P3TCK_02186-P3TCK_02126) that encodes for the alpha (COG0804), beta (COG0832) and gamma (COG0831) subunits of urease and its accessory proteins. These genes are arranged in a single operon that also encoded for an ABC transporter. Their sequences did not display an altered GC%, GC skew or codon usage suggesting that they had been lost by SS9 rather than acquired by 3TCK. Nevertheless, their presence might be reflective of differences in the chemistry of the more eutrophic habitat of the San Diego Bay sediments (where *P. profundum* 3TCK was isolated) versus that associated with deep-sea amphipods (from which *P. profundum* SS9 was isolated) and is a further evidence of how the genome plasticity of *P. profundum* is the key to its adaptive radiation.

### The Conversion of the Deep Bathytype to UV Resistance

The absence of light (apart from chemiluminescence or possibly bioluminescence) in the deepest depths of the oceans argues for a selective loss of genes associated with light tolerance. In fact the piezophile *Psychromonas* species strain CNPT3 [51] has been shown to be extremely sensitive to UV radiation [52].

The two most common types of UV-induced lesions on DNA are the generation of cyclobutane pyrimidine dimers (CPDs) and pyrimidine-pyrimidone 6–4 photoproducts (6–4PPs) [53]. This damage to DNA can be repaired by multiple pathways [53], but photoreactivation by deoxyribodipyrimidine photolyase, the product of the *phr* gene, is unique in that it requires blue light energy to split the CPDs or the 6–4PPs [54].

Because of this blue light requirement the genes for the deoxyribodipyrimidine photolyase are expected to be absent from the genomes of deep-sea microbes. In fact, the SS9 genome does not contain a *phr* gene and the metagenomic analysis of the distribution of genes in a stratified water column [47] showed significant over-representation of *phr* genes from the photic region when compared to samples from deeper waters.

In contrast to SS9, other members of the family Vibrionaceae have been shown to primarily rely on the activity of photolyases for the repair on UV-induced damage [55], [56]. The genome of *Vibrio cholerae* N16961 [57] encodes for three different members of the cryptochrome/photolyase family [56]. The first one (VCA0057) functions in repairing CPDs in dsDNA [56] the second one (VC1814) in repairing CPDs in ssDNA [55] while the function of the third one (VC1392) is still unknown. A similar array of photolyase-like ORFs can be seen in the genomes most other members of the Vibrionaceae, including the draft genome of *Photobacterium* sp. SKA34 (https://moore.jcvi.org/moore/SingleOrganism.do?speciesTag=SKA34) encoding for orthologs to all three cryptochromes/photolyases of *V. cholerae*.

**Figure 5:**
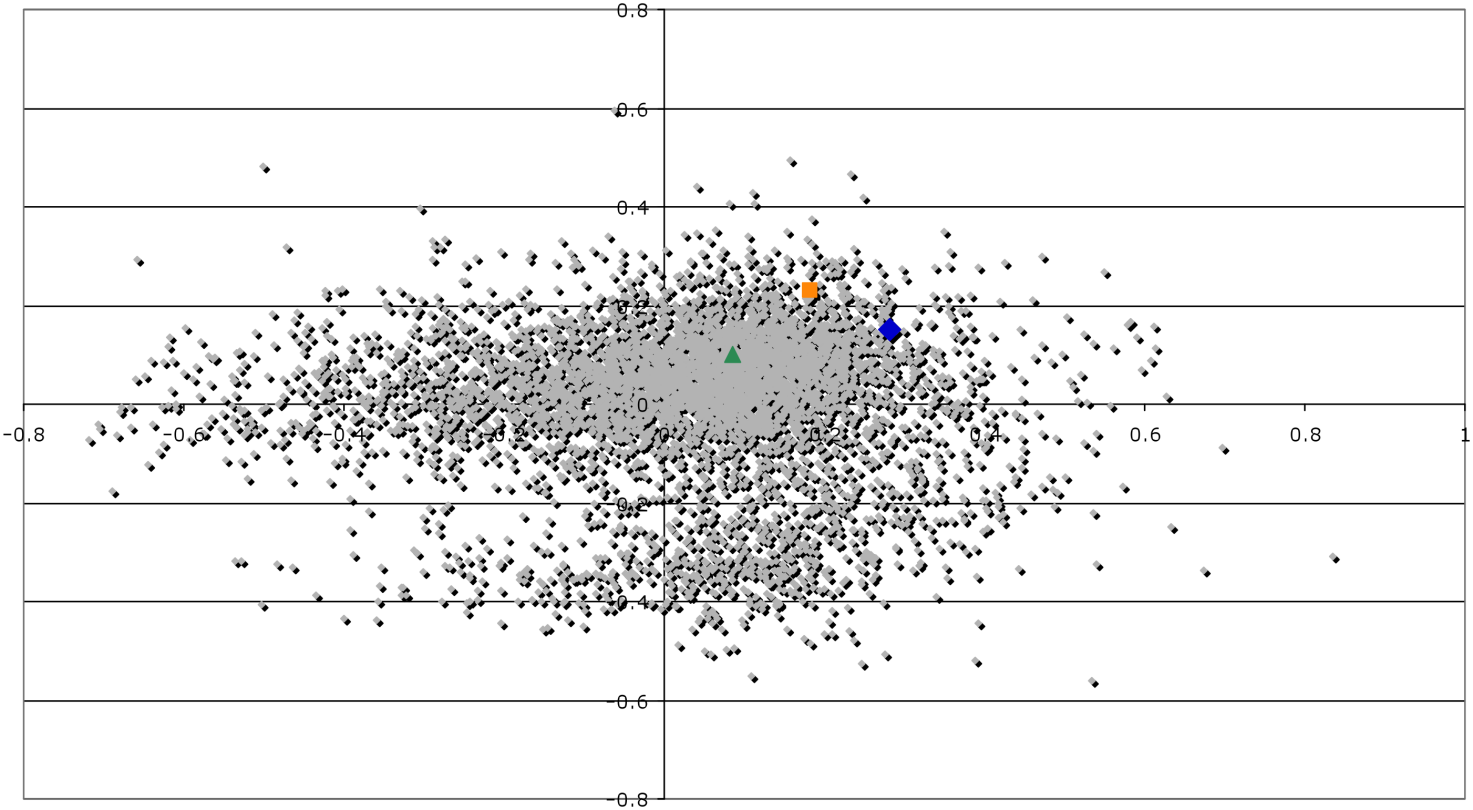
Correspondance analysis of codon usage for the ORFs of *P. profundum* 3TCK. The ORFs belonging to the *phr* gene cluster (in the insert) are color coded. The green triangle represents P3TCK_10668, the yellow square P3TCK_10673, and the blue diamond P3TCK_10663. The codon usage of P3TCK_10663 and P3TCK_10673 suggests their recent acquisition by horizontal gene transfer.

The shallow bathytype 3TCK contains a *phr* gene within a three gene cluster (P3TCK_10663, P3TCK_10668, P3TCK10673) on chromosome 2 with altered codon usage (Figure 5). The *phr* gene (P3TCK_10663) and the upstream hypothetical protein (P3TCK_10668) are part of a predicted operon with a promoter upstream of P3TCK_10668 driving their expression. A different promoter in the opposite direction drives the transcription of *rpoX* (P3TCK_10673), an ECF-type sigma factor. Because of this arrangement, it was hypothesised that the *phr* gene cluster had been acquired by HGT under the selective forces provided by UV light exposure in shallow water.

**Figure 6:**
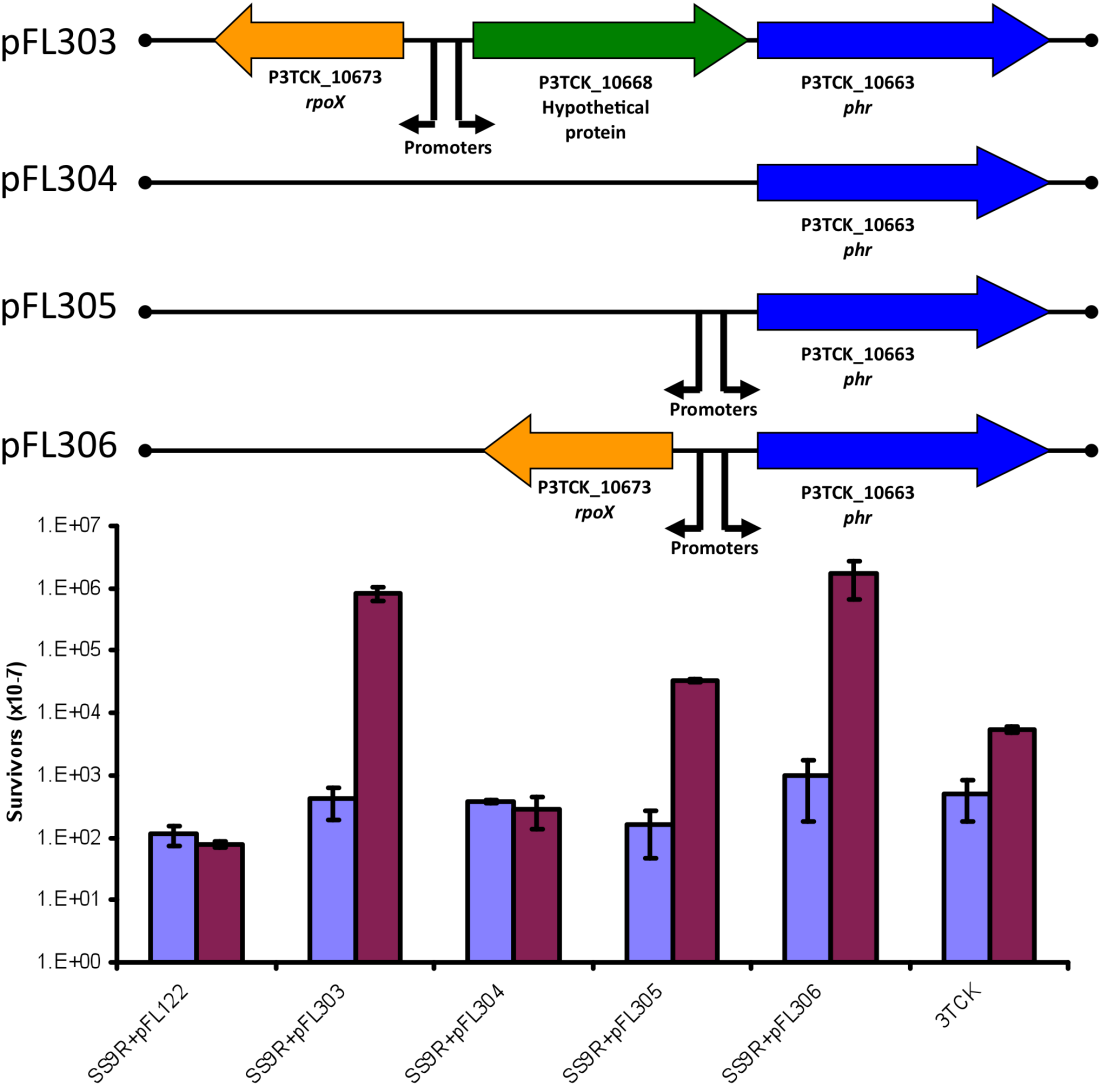
Introduction of the *phr* gene cluster from the shallow bathytype 3TCK into the deep bathytype SS9 confers UV resistance. This phenotype is not observed in the deletion construct that lacks the upstream hypothetical protein and *rpoX* gene (pFL304). The UV resistance phenotype can be partially restored by re-adding to pFL304 the promoter region of the cluster (pFL305) but is completely restored only when both the promoter and the *rpoX* sigma factor are added (pFL306). The absence of P3TCK_10668 (Hypothetical Protein) does not affect UV sensitivity. The UV survival plots present the ratio of c.f.u. observed after UV exposure followed by blue-light photoreactivation (red) compared to the non-photoreactivated controls (blue) as described in the materials and methods.

The cluster was cloned on a broad host-range plasmid and introduced into SS9 (Figure 6; pFL303) resulting in approximately 1,000 fold increase in colony forming units (c.f.u.) survival after UV irradiation compared to the controls. This survival was dependant on blue-light incubation (Figure 6). A deletion encompassing *rpoX* and most of the hypothetical protein (P3TCK_10668) abolished photoreactivation (pFL304). If the promoter region is re-introduced in the right orientation into the deletion construct (pFL305), photoreactivation was partially restored, yielding approximately 100-fold more surviving c.f.u. than the untreated controls. The full restoration of the UV resistant phenotype could be obtained only by cloning, in the right orientation, both the promoter and the sigma factor (pFL306).

**Figure 7:**
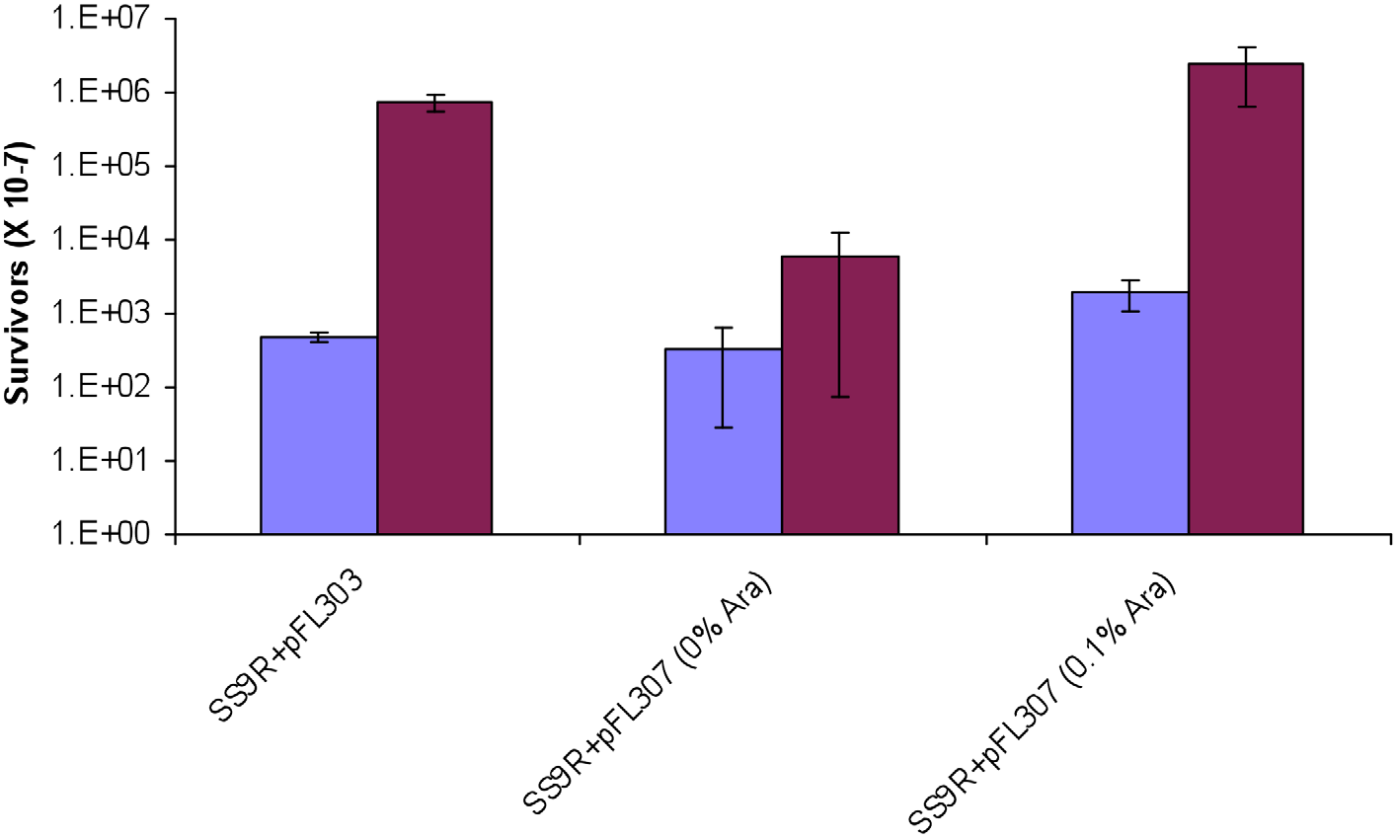
The UV resistant phenotype depends uniquely on the levels of expression of the *phr* gene (P3TCK_10673). Cloning of the *phr* gene under the arabinose-inducible promoter of pFL190 confers UV-resistance to the cells only when grown with 0.1% L-arabinose. The UV survival plots present the ratio of c.f.u. observed after UV exposure followed by blue-light photoreactivation (red) compared to the non-photoreactivated controls (blue) as described in the materials and methods.

Based upon these results it was suggested that the gene encoding the hypothetical protein is dispensable for photoreactivation activity and the UV resistance phenotype is solely dependent on the level of expression of the *phr* gene. To test both hypotheses the *phr* gene alone was cloned in a vector (pFL190) with an arabinose-inducible promoter (pFL307). The full UV resistant phenotype was observed only after induction with 0.1% arabinose (Figure 7) indicating that high levels of expression of the *phr* gene alone are necessary and sufficient to confer UV resistance.

A noteworthy result of this experiment is that the functional activity of the *phr* gene cluster benefits from the presence of a flanking sigma factor. There is a precedent for this type of observation. Sometimes acquired genes must be obtained as clusters of functional units to overcome the barrier caused by the incapacity to transcribe the HGT gene at the appropriate level [58]. Genes providing marginal benefits, like photolyase, can also be readily lost from a population when the increased metabolic cost for replication is not balanced by selective pressure as was observed between high- and low-light-adapted *Prochlorococcus* strains [59]. These processes of gene cluster gain and loss generate and maintain the genomic diversity within bathytypes while restricting their niche.

## References

[1] Witte U, Wenzhofer F, Sommer S, Boetius A, Heinz P, et al (2003) In situ experimental evidence of the fate of a phytodetritus pulse at the abyssal sea floor. Nature 424: 763–766 10.1038/nature01799 12917681

[2] Sogin ML, Morrison HG, Huber JA, Welch DM, Huse SM, et al (2006) Microbial diversity in the deep sea and the underexplored “rare biosphere”. Proc Natl Acad Sci USA 103: 12115–12120 10.1073/pnas.0605127103 16880384 PMC1524930

[3] Eloe EA, Fadrosh DW, Novotny M, Zeigler Allen L, Kim M, et al (2011) Going Deeper: Metagenome of a Hadopelagic Microbial Community. PLoS ONE 6: e20388 10.1371/journal.pone.0020388 21629664 PMC3101246

[4] Brown MV, Philip GK, Bunge JA, Smith MC, Bissett A, et al (2009) Microbial community structure in the North Pacific ocean. ISME J 3: 1374–1386 10.1038/ismej.2009.86 19626056

[5] Lauro FM, Bartlett DH (2007) Prokaryotic lifestyles in deep sea habitats. Extremophiles 12: 15–25 10.1007/s00792-006-0059-5 17225926

[6] Eloe EA, Malfatti F, Gutierrez J, Hardy K, Schmidt WE, et al (2011) Isolation and characterization of a psychropiezophilic Alphaproteobacterium. Appl Environ Microbiol 77: 8145–8153 10.1128/AEM.05204-11 21948832 PMC3208983

[7] Khelaifia S, Fardeau ML, Pradel N, Aussignargues C, Garel M, et al (2011) *Desulfovibrio piezophilus*, sp. nov., a novel piezophilic sulfate-reducing bacterium isolated from wood falls in Mediterranean Sea. Int J Syst Evol Microbiol 61: 2706–2711 10.1099/ijs.0.028670-0 21169465

[8] Bale SJ, Goodman K, Rochelle PA, Marchesi JR, Fry JC, et al (1997) *Desulfovibrio profundus* sp. nov., a novel barophilic sulphate-reducing bacterium from deep sediment layers in the Japan Sea. Int J Syst Bacteriol 47: 515–521 10.1099/00207713-47-2-515 9103642

[9] Alain K, Marteinsson VT, Miroshnichenko ML, Bonch-Osmolovskaya EA, Prieur D, et al (2002) *Marinitoga piezophila* sp. nov., a rod-shaped, thermo-piezophilic bacterium isolated under high hydrostatic pressure from a deep-sea hydrothermal vent. Int J Syst Evol Microbiol 52: 1331–1339 10.1099/ijs.0.02068-0 12148648

[10] Lauro FM, Chastain RA, Blankenship LE, Yayanos AA, Bartlett DH (2007) The unique 16S rRNA genes of piezophiles reflect both phylogeny and adaptation. Appl Environ Microbiol 73: 838–845 10.1128/AEM.01726-06 17158629 PMC1800765

[11] Vezzi A, Campanaro S, D’Angelo M, Simonato F, Vitulo N, et al (2005) Life at depth: *Photobacterium profundum* genome sequence and expression analysis. Science 307: 1459–1461 10.1126/science.1103341 15746425

[12] Campanaro S, Vezzi A, Vitulo N, Lauro FM, D’Angelo M, et al (2005) Laterally transferred elements and high pressure adaptation in *Photobacterium profundum* strains. BMC Genomics 6: 122 10.1186/1471-2164-6-122 16162277 PMC1239915

[13] Campanaro S, Pascale FD, Telatin A, Schiavon R, Bartlett DH, et al (2012) The transcriptional landscape of the deep-sea bacterium *Photobacterium profundum* in both a toxR mutant and its parental strain. BMC Genomics 13: 567 10.1186/1471-2164-13-567 23107454 PMC3505737

[14] DeLong EF, Franks DG, Yayanos AA (1997) Evolutionary relationships of cultivated psychrophilic and barophilic deep-sea bacteria. Appl Environ Microbiol 63: 2105–2108. 16535621 10.1128/aem.63.5.2105-2108.1997 PMC1389176

[15] Nogi Y, Masui N, Kato C (1998) *Photobacterium profundum* sp. nov., a new, moderately barophilic bacterial species isolated from a deep-sea sediment. Extremophiles 2: 1–7. 9676237 10.1007/s007920050036

[16] Biddle JF, House CH, Brenchley JE (2005) Enrichment and cultivation of microorganisms from sediment from the slope of the Peru Trench (ODP Site 1230). In: Jørgensen BB, D’Hondt SL, Miller DJ (Eds.) Proc ODP Sci Results 201: 1–19.

[17] Wilkins D, van Sebille E, Rintoul SR, Lauro FM, Cavicchioli R (2013) Advection shapes Southern Ocean microbial assemblages independent of distance and environment effects. Nat Commun 4: 2457 10.1038/ncomms3457 24036630

[18] Vanlint D, Mitchell R, Bailey E, Meersman F, McMillan PF, et al (2011) Rapid acquisition of gigapascal-high-pressure resistance by *Escherichia coli* . mBio 2: e00130–10 10.1128/mBio.00130-10 21264062 PMC3025523

[19] Hutchinson GE (1957) Concluding remarks. Cold Spring Harbor Symposia on Quantitative Biology 22: 415–427.

[20] Yayanos AA, Vanboxtel R (1982) Coupling device for quick high-pressure connections to 100 Mpa. Review of Scientific Instruments 53: 704–705.

[21] Yayanos AA (2001) Deep-sea piezophilic bacteria. In: J. Paul (ed.), Marine Microbiology. Academic Press p. 615–638.

[22] Chi E, Bartlett DH (1993) Use of a reporter gene to follow high-pressure signal-transduction in the deep-sea bacterium *Photobacterium* sp. strain SS9. J Bacteriol 175: 7533–7540. 8244922 10.1128/jb.175.23.7533-7540.1993 PMC206909

[23] Goldberg SMD, Johnson J, Busam D, Feldblyum T, Ferriera S, et al (2006) A Sanger/pyrosequencing hybrid approach for the generation of high-quality draft assemblies of marine microbial genomes. Proc Natl Acad Sci USA 103: 11240–11245 10.1073/pnas.0604351103 16840556 PMC1544072

[24] Huson DH, Reinert K, Kravitz SA, Remington KA, Delcher AL, et al (2001) Design of a compartmentalized shotgun assembler for the human genome. Bioinformatics 17: S132–S139 10.1093/bioinformatics/17.suppl_1.S132 11473002

[25] Nakasone K, Masui N, Takaki Y, Sasaki R, Maeno G, et al (2000) Characterization and comparative study of the rrn operons of alkaliphilic *Bacillus halodurans* C-125. Extremophiles 4: 209–214 10.1007/PL00010713 10972189

[26] Allen MA, Lauro FM, Williams TJ, Burg D, Siddiqui KS, et al (2009) The genome sequence of the psychrophilic archaeon, *Methanococcoides burtonii*: the role of genome evolution in cold adaptation. ISME J 3: 1012–1035 10.1038/ismej.2009.45 19404327

[27] Rodriguez-Brito B, Rohwer F, Edwards RA (2006) An application of statistics to comparative metagenomics. BMC Bioinformatics. 7: 162 10.1186/1471-2105-7-162 PMC1473205 16549025

[28] Goris J, Konstantinidis KT, Klappenbach JA, Coenye T, Vandamme P, et al (2007) DNA-DNA hybridization values and their relationship to whole-genome sequence similarities. Int J Syst Evol Microbiol 57: 81–91 10.1099/ijs.0.64483-0 17220447

[29] Wall DP, Deluca T (2007) Ortholog detection using the reciprocal smallest distance algorithm. Methods Mol Biol 396: 95–110 10.1007/978-1-59745-515-27 18025688

[30] Edgar RC (2004) MUSCLE: multiple sequence alignment with high accuracy and high throughput. Nucl. Acids Res 32: 1792–1797 10.1093/nar/gkh340 PMC390337 15034147

[31] Zhang Z, Li J, Zhao XQ, Wang J, Wong GK, et al (2006) KaKs Calculator: Calculating Ka and Ks through model selection and model averaging. Genomics Proteomics Bioinformatics 4: 259–263 10.1016/S1672-0229(07)60007-2 17531802 PMC5054075

[32] Yang Z, Nielsen R (2000) Estimating synonymous and nonsynonymous substitution rates under realistic evolutionary models. Mol Biol Evol 17: 32–43. 10666704 10.1093/oxfordjournals.molbev.a026236

[33] Mutreja A, Kim DW, Thomson NR, Connor TR, Lee JH, et al (2011) Evidence for multiple waves of global transmission within the seventh cholera pandemic. Nature 477: 462–465 10.1038/nature10392 21866102 PMC3736323

[34] Karlin S, Mrazek J, Campbell AM (1998) Codon usages in different gene classes of the *Escherichia coli* genome. Mol Microbiol 29: 1341–1355. 9781873 10.1046/j.1365-2958.1998.01008.x

[35] Davis JJ, Olsen GJ (2010) Modal codon usage: assessing the typical codon usage of a genome. Mol Biol Evol 27: 800–810. 20018979 10.1093/molbev/msp281 PMC2839124

[36] Davis JJ, Olsen GJ (2011) Characterizing native codon usages of a genome: an axis projection approach. Mol Biol Evol 28: 211–221. 20679093 10.1093/molbev/msq185 PMC3002238

[37] Lauro FM, Eloe EA, Liverani N, Bertoloni G, Bartlett DH (2005) Conjugal vectors for cloning, expression, and insertional mutagenesis in gram-negative bacteria. Biotechniques 38: 708–712. 15945369 10.2144/05385BM06

[38] Tamburini C, Garcin J, Gregori G, Leblanc K, Rimmelin P, et al (2006) Pressure effects on surface Mediterranean prokaryotes and biogenic silica dissolution during a diatom sinking experiment. Aquat Microb Ecol 43: 267–276 10.3354/ame043267

[39] Bartlett DH (2002) Pressure effects on in vivo microbial processes. Biochim Biophys Acta 1595: 367–381. 11983409 10.1016/s0167-4838(01)00357-0

[40] Le Bihan T, Rayner J, Roy MM, Spagnolo L (2013) *Photobacterium profundum* under pressure: a MS-based label-free quantitative proteomics study. PLoS ONE 8: e60897 10.1371/journal.pone.0060897 23741291 PMC3669370

[41] Egan ES, Waldor MK (2003) Distinct replication requirements for the two *Vibrio cholerae* chromosomes. Cell 114: 521–530 10.1016/S0092-8674(03)00611-1 12941279

[42] Okada K, Iida T, Kita-Tsukamoto K, Honda T (2005) Vibrios commonly possess two chromosomes. J Bacteriol 187: 752–757 10.1128/JB.187.2.752-757.2005 15629946 PMC543535

[43] Shulse CN, Allen EE (2011) Widespread occurrence of secondary lipid biosynthesis potential in microbial lineages. PLoS ONE 6: e20146 10.1371/journal.pone.0020146 21629834 PMC3098273

[44] Větrovský T, Baldrian P (2013) The variability of the 16S rRNA gene in bacterial genomes and its consequences for bacterial community analyses. PLoS ONE 8: e57923 10.1371/journal.pone.0057923 23460914 PMC3583900

[45] Pei A, Nossa CW, Chokshi P, Blaser MJ, Yang L, et al (2009) Diversity of 23S rRNA Genes within Individual Prokaryotic Genomes. PLoS ONE 4: e5437 10.1371/journal.pone.0005437 19415112 PMC2672173

[46] Carver TJ, Rutherford KM, Berriman M, Rajandream MA, Barrell BG, et al (2005) ACT: the Artemis Comparison Tool. Bioinformatics 21: 3422–3423 10.1093/bioinformatics/bti553 15976072

[47] DeLong EF, Preston CM, Mincer T, Rich V, Hallam SJ, et al (2006) Community genomics among stratified microbial assemblages in the ocean’s interior. Science 311: 496–503 10.1126/science.1120250 16439655

[48] Lauro FM, Tran K, Vezzi A, Vitulo N, Valle G, et al (2008) Large-scale transposon mutagenesis of *Photobacterium profundum* SS9 reveals new genetic loci important for growth at low temperature and high pressure. J Bacteriol 190: 1699–1709 10.1128/JB.01176-07 18156275 PMC2258685

[49] McCarter LL (2004) Dual flagellar systems enable motility under different circumstances. J. Mol. Microbiol. Biotechnol. 7: 18–29 10.1159/000077866 15170400

[50] Eloe EA, Lauro FM, Vogel RF, Bartlett DH (2008) The deep-sea bacterium *Photobacterium profundum* SS9 utilizes separate flagellar systems for swimming and swarming under high-pressure conditions. Appl Environ Microbiol 74: 6298–6305 10.1128/AEM.01316-08 18723648 PMC2570297

[51] Lauro FM, Stratton TK, Chastain RA, Ferriera S, Johnson J, et al (2013) Complete Genome Sequence of the Deep-Sea Bacterium *Psychromonas* Strain CNPT3. Genome Announc 1: e00304–13 10.1128/genomeA.00304-13 23723403 PMC3668011

[52] Lutz L, Yayanos AA (1986) UV Repair in Deep-Sea Bacteria. Federation Proceedings 45: 1784–1784.

[53] Yasui A, McCready SJ (1998) Alternative repair pathways for UV-induced DNA damage. Bioessays 20: 291–297. 9619100 10.1002/(SICI)1521-1878(199804)20:4<291::AID-BIES5>3.0.CO;2-T

[54] Todo T (1999) Functional diversity of the DNA photolyase/blue light receptor family. Mutat Res 434: 89–97. 10422537 10.1016/s0921-8777(99)00013-0

[55] Selby CP, Sancar A (2006) A cryptochrome/photolyase class of enzymes with single-stranded DNA-specific photolyase activity. Proc Natl Acad Sci USA 103: 17696–17700 10.1073/pnas.0607993103 17062752 PMC1621107

[56] Worthington EN, Kavakli IH, Berrocal-Tito G, Bondo BE, Sancar A (2003) Purification and characterization of three members of the photolyase/cryptochrome family blue-light photoreceptors from *Vibrio cholerae* . J Biol Chem 278: 39143–39154 10.1074/jbc.M305792200 12878596

[57] Heidelberg JF, Eisen JA, Nelson WC, Clayton RA, Gwinn ML, et al (2000) DNA sequence of both chromosomes of the cholera pathogen *Vibrio cholerae* . Nature 406: 477–483 10.1038/35020000 10952301 PMC8288016

[58] Kurland CG, Canback B, Berg OG (2003) Horizontal gene transfer: a critical view. Proc Natl Acad Sci USA 100: 9658–9662 10.1073/pnas.1632870100 12902542 PMC187805

[59] Rocap G, Larimer FW, Lamerdin J, Malfatti S, Chain P, et al (2003) Genome divergence in two *Prochlorococcus* ecotypes reflects oceanic niche differentiation. Nature 424: 1042–1047 10.1038/nature01947 12917642

[60] Murray NE, Brammar WJ, Murray K (1977) Lambdoid phages that simplify the recovery of in vitro recombinants. Mol Gen Genet 150: 53–61. 319344 10.1007/BF02425325

[61] Hanahan D (1983) Studies on transformation of *Escherichia coli* with plasmids. J Mol Biol 166: 557–580. 6345791 10.1016/s0022-2836(83)80284-8

[62] Better M, Helinski DR (1983) Isolation and characterization of the *recA* gene of *Rhizobium meliloti* . J Bacteriol 155: 311–316. 6305915 10.1128/jb.155.1.311-316.1983 PMC217682

